# Effects of Blended-Cement Paste Chemical Composition Changes on Some Strength Gains of Blended-Mortars

**DOI:** 10.1155/2014/625350

**Published:** 2014-01-22

**Authors:** Mehmet Serkan Kirgiz

**Affiliations:** Civil Engineering Department, Engineering Faculty, Hacettepe University, Beytepe, 06800 Ankara, Turkey

## Abstract

Effects of chemical compositions changes of blended-cement pastes (BCPCCC) on some strength gains of blended cement mortars (BCMSG) were monitored in order to gain a better understanding for developments of hydration and strength of blended cements. Blended cements (BC) were prepared by blending of 5% gypsum and 6%, 20%, 21%, and 35% marble powder (MP) or 6%, 20%, 21%, and 35% brick powder (BP) for CEMI42.5N cement clinker and grinding these portions in ball mill at 30 (min). Pastes and mortars, containing the MP-BC and the BP-BC and the reference cement (RC) and tap water and standard mortar sand, were also mixed and they were cured within water until testing. Experiments included chemical compositions of pastes and compressive strengths (CS) and flexural strengths (FS) of mortars were determined at 7th-day, 28th-day, and 90th-day according to TS EN 196-2 and TS EN 196-1 present standards. Experimental results indicated that ups and downs of silica oxide (SiO_2_), sodium oxide (Na_2_O), and alkali at MP-BCPCC and continuously rising movement of silica oxide (SiO_2_) at BP-BCPCC positively influenced CS and FS of blended cement mortars (BCM) in comparison with reference mortars (RM) at whole cure days as MP up to 6% or BP up to 35% was blended for cement.

## 1. Introduction

Increasing of marble and brick manufacturing and lack of suitable landfills for wastes and ineffective purification systems for marble waste (MW) and brick waste (BW) have resulted in a move to raise both volume of these wastes and loss of the useful minerals. MW and BW occur, respectively, at 2,592,000 (tons) and 3,800,000 (tons) in Turkey every year as like whole world [1].

Activation of cement, such byproducts as fly ash (FA), silica fume (SF), MP, and BP, has been appealed by researchers for more than ten decades. Several researches have presented benefits of byproducts replaced by sand in mortar and concrete such properties as strength and workability [2–17]. Compatibility of byproducts for cement and mortar is assessed according to standard methods and technological studies (XRD, SEM, EDS, and TEM) and equations suggested by researchers to compute C_3_S, C_2_S, C_3_A, and C_4_AF compounds in cement [18–25]. Suggested standards, technological studies, and equations are inadequate to explain effects of blended-cement paste chemical composition changes on some strength gains of blended-mortars. Compounds linked strength gain at hydrated cement pastes are C_3_S, C_2_S, C_3_A, and C_4_AF are composed of CaO, SiO_2_, Al_2_O_3_, and Fe_2_O_3_ known major oxides. Basically, these major oxides underlie the main reason for which cements show strength gain quickly (false strength) or strength gain slowly. However, CaO and SiO_2_ of OPC pastes at 28 d were, respectively, over 9% greater and over 6% less than that of OPC at 7 d. Due to these changes of CaO and SiO_2_, OPC mortars at 28th-d showed over 25% greater quick strength gain in comparison with OPC mortars at 7th-d (Tables 3, 4, 5, and 6). This strength gain tendency of cement was considered to be able to change chemical compositions in which cement paste is hydrated by C_3_S, C_2_S, C_3_A and C_4_AF.

Therefore, this research was planned to monitor the effects of blended-cement paste chemical composition changes on some strength gains of blended mortars. This paper does not address either the properties of concrete made with MP and BP or porosity effect for mortar strength gain or CaCl_2_ leaching effects on strength gains of mortars.

## 2. Experimental Procedures

### 2.1. Materials

Marble waste (MW), brick waste (BW), CEMI42.5N clinker, gypsum, and tap water were used for materials in this up-to-date experimental research. MW and BW were collected from plants which were found in Bilecik province and Kaman town in Turkey, respectively. CEMI42.5N clinker and gypsum were sent out SET Cement Factory in Ankara province. Marble waste or brick waste was granulated at thirty minutes in laboratory ball mill in order to make them a powder form (MP, BP) [1]. Chemical composition of MP composed of 53.79% CaO, 0.388% SiO_2_, 0.11% Al_2_O_3_, 0.04% Fe_2_O_3_, 0.77% MgO, 0.05% SO_3_, 0.01% K_2_O, 0.34% Na_2_O, and 44.15% loss on ignition (LOI). Chemical composition of BP consisted of 12.88% CaO, 39.55% SiO_2_, 15.71% Al_2_O_3_, 14.05% Fe_2_O_3_, 3.29% MgO, 0.48% SO_3_, 1.98% K_2_O, 0% Na_2_O, and 11.30% LOI [1]. Chemical composition of CEMI42.5N included 65.09% CaO, 21.01% SiO_2_, 5.61% Al_2_O_3_, 3.52% Fe_2_O_3_, 1.55% MgO, 0.87% SO_3_, 0.86% K_2_O, 0.81% Na_2_O, and 0.30% LOI [26]. Because of MP's and BP's invaluable chemical compositions, assortment of MP is early strength gain activator and type of BP is unnatural pozzolana for cement, mortar, and concrete [1, 19, 27]. Physical and pozzolanic properties of MP, BP and physical properties of CEMI42.5N cement are shown in Table 1 [1].

MW or BW can also be partially added for CEMI42.5 cement clinker and gypsum in order to positively improve chemical compositions of cement pastes and strength gains of blended mortars according to physical and activation properties of MP and BP (Table 1) [1].

**Figure 1:**
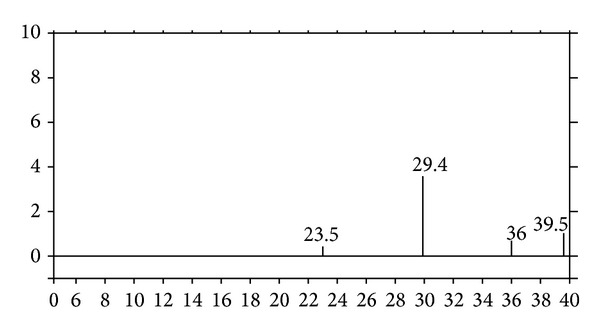
XRD diagram of MP [1].

Mineralogical properties of MP measured by XRD are given in Figure 1. MP mainly contains middle pure level-calcite (CaCO_3_). There are found some other minor minerals, for instance, quartz, dolomite, hematite, and magnetite in XRD pattern of MP (Figure 1) [1].

**Figure 2:**
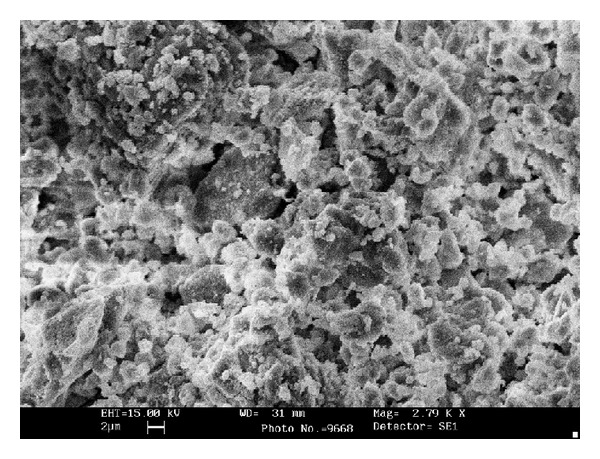
SEM image of MP microstructure [1].

**Figure 3:**
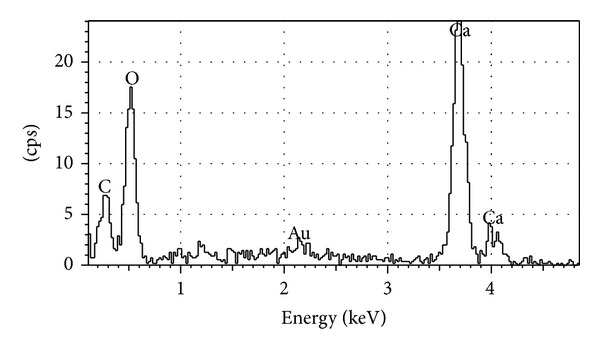
Element counts of MP [1].

Figure 2 showed that most of the MP particles consisted of near-spherical and spheres were quite smooth. It is also apparent that MP is composed of various sized particles ranging from several to dozens of ultrafine micrometers. Additionally, microstructure of MP has slightly little spherical and amorphous hollow (Figure 2). There are mainly Ca, C, and O element peaks in EDS of MP similar to its chemical composition (Figure 3). As measured through absorbance spectra, results show that MP turned out big and little annular and sphere particle [1].

**Figure 4:**
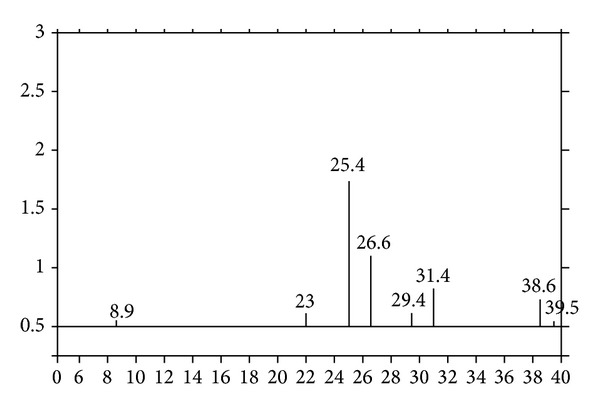
XRD diagram of BP [1].

Figure 4 shows X-ray diffraction of BP. Characteristic mineralogical properties of BP consist of silica oxide (SiO_2_), aluminum oxide (Al_2_O_3_), magnesium orthosilicate (2[Mg_0,96_Fe_0,04_]O·SiO_2_), and magnesium silicate (Mg_2_SiC_4_) (Figure 4) [1].

**Figure 5:**
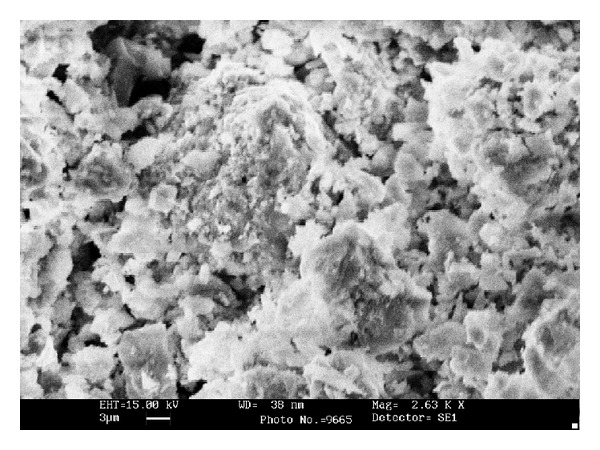
SEM image of BP microstructure [1].

**Figure 6:**
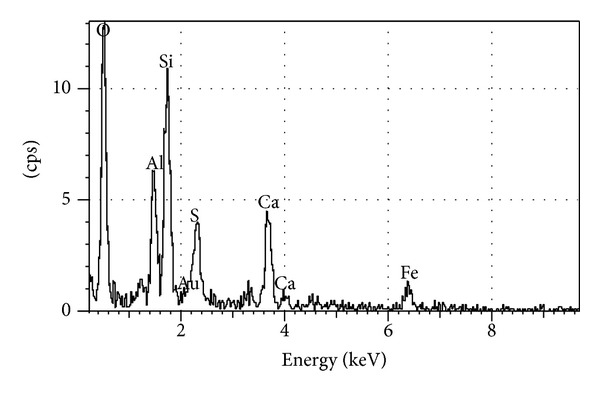
Element counts of BP [1].

SEM images of BP and EDS element counts of BP are shown in Figures 5 and 6, respectively. SEM observations of BP shown in Figure 5 indicated amorphous structure of BP. As measured through absorbance spectra, results show that BP turned out big and little annular and flat particle. Grains of BP exhibit irregular shapes with flat surfaces covered by small debris which are similar cement grains in paste and mortar. BP samples also contain immature needle-like products. It is apparent that populations of these products in the BP are significantly higher than that of the MP and these products are more compacted, as well (Figure 5) [1]. On the contrary to MP observations, BP includes plenty of rich silicate, aluminate, calcite, and hematite minerals which reduce detrimental effects and provide to have much stable chemical compositions for cement. There are mainly Ca, Si, Fe, S, and Al element peaks in EDS of BP similar to its chemical composition (Figure 6) [1].

### 2.2. Sample Preparation

Difference of this research than others is that sample preparation consists of three stages. First stage is preparation of blended cements. Blended cements were prepared by blending of MW or BW for CEMI42.5N cement clinker at the percents of 6, 20, 21, and 35 and adding gypsum up to 5%, and these mixture proportions were grinded in ball mill together at 30 min. Blending ratios of MW, BW, and gypsum for cement clinker were chosen according to TS EN 196-1 in order to be appropriate for this up-to-date standard. Codes of cement samples and mixture proportions and grinding time are given in Table 2 [1].

Second stage is preparation of cement pastes containing MP-blended cements or BP-blended cements or reference cement in order to measure chemical composition changes at 7th-d, 28th-d, and 90th-d. A medium planetary mixer was used for every paste mix using this following procedure: (1) add water and cements in bowl and mix them for 90 s at low speed; (2) stop mixer for 15 s to scrape bowl; mix for another 90 s at high speed; (3) cast pastes in cubic mold 50 mm for 4 min totally. Pastes were mixed with water : cement ratio of 1 : 2 [1, 26, 28].

Last stage deals with preparation of mortars containing MP-blended cements or BP-blended cements or reference cement for monitoring gains of CS and FS at 7th-d, 28th-d, and 90th-d. In preparation of mortars, a medium planetary mixer was used for 4 min following procedure: (1) add water and cement in bowl; (2) mix them for 30 s at low speed; (3) add CEN standard sand at 30 s; (4) mix them for 30 s at high speed; (5) stop the mixer for 15 s to scrape bowl; (6) mix for 60 s at high speed; (7) cast each mortar mixes in prism mould 40 × 40 × 160 mm as three layers; (8) collapse each layer 60 times [1, 28]. Mortars were mixed with water : cement : sand ratio of 1 : 2 : 6. Codes of blended cement mortars, reference cement mortars, and mixture proportions of mortars for one standard 3-gang mould are presented in Table 3 [1].

### 2.3. Methods

Experimental programs had three main goals: observe changes of some blended-cement paste morphology; analyze chemical composition changes of blended-cement pastes; monitor gains of compressive strength and flexural strength of mortars.

#### 2.3.1. Morphology of Pastes

Some mortars continued strength gain at 7th-d, 28th-d, and 90th-d although silica based chemical compositions of pastes went down. Therefore, CEMI42.5MP6P and CEMI42.5NBP20P pastes were observed in SEM equipped *energy dispersive spectroscope* (EDS) in order to better explain strength developments which were associated with C-S-H gel, Ca(OH)_2_, ettringite, C-A-S, and unhydrated cement particles. All samples were coated with gold. SEM was set so the upper detector collected the secondary electrons. SEM observations were performed on these samples using a LEO scanning electron microscope.

#### 2.3.2. Chemical Compositions Experiment (with Wet Analysis)

Blended-cement pastes and reference cement pastes were dried in laboratory and grinded at the end of curing days of the 7-day, 28-day, and 90-day ages. TS EN 196-2 standard was known wet chemical analysis was followed to analyze the chemical compositions of the pastes [26]. After 24 h casting in plastic mould in automatic controlled curing cabinet at 98% relative humidity, these pastes were subjected to water curing in the same cure cabinet until testing at 21°C and 98% relative humidity. For each mix at each age, three samples were analyzed and the average value of 162 samples was taken to be the representative chemical properties.

#### 2.3.3. Compressive Strength (CS) Experiment

CSs of mortars were determined cubic samples which were used portions of prisms broken in flexural strength experiment at 7 d, 28 d, and 90 d in accordance to standard method [28]. For each mix at each age, twelve samples were tested with an ELE hydraulic testing machine and the average value of 324 samples was taken to be the representative strength.

#### 2.3.4. Flexural Strength (FS) Experiment

Standard method was followed to measure flexural strength of prism mortar samples 40 × 40 × 160 mm with one-point loading at 7 d, 28 d, and 90 d. After 1 day of casting, samples were demolded and cured in water in automatic controlled curing cabinet at 21°C and 98% relative humidity until testing. Curing water was refreshed fortnightly [28]. For each mix at each age, six samples were tested with an ELE hydraulic testing machine and the average value of 162 samples was taken to be the descriptive strength.

## 3. Results and Discussions

### 3.1. Influences of the BCP-SEM Image Changes to CS and FS of Blended Cement Mortars (BCM)

**Figure 7:**
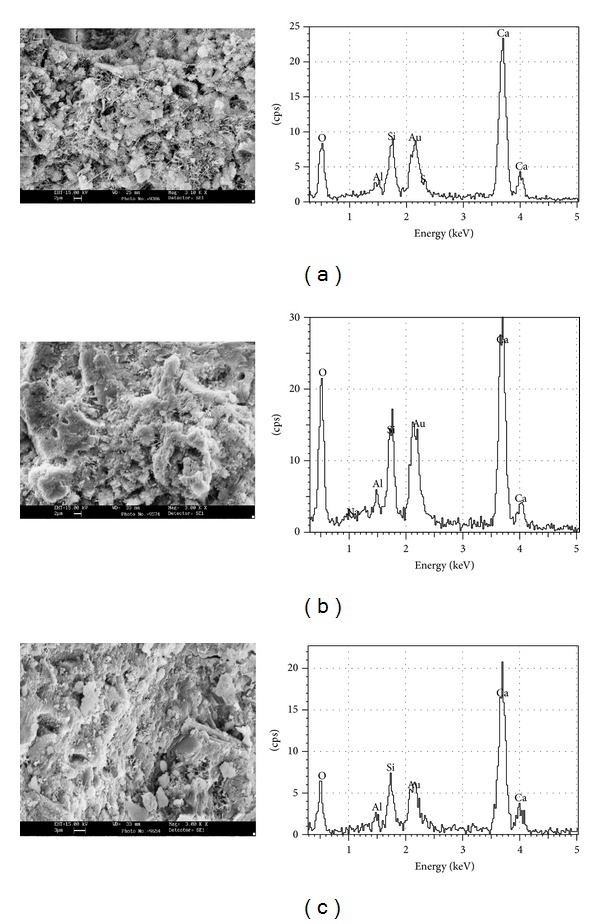
SEM images and EDS graphs observed for CEMI42.5MP6P at 7 d, 28 d, and 90 d.

**Figure 8:**
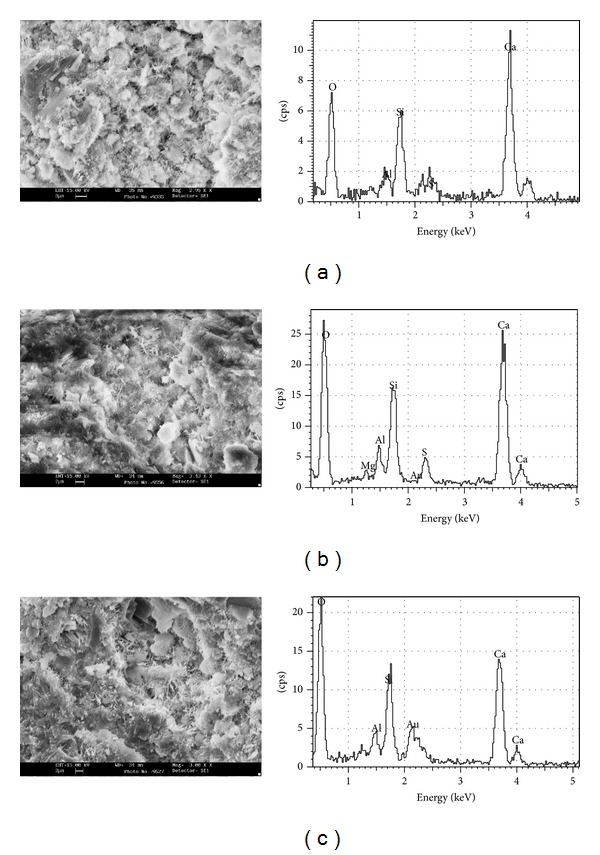
SEM images and EDS graphs observed for CEMI42.5BP20P at 7 d, 28 d, and 90 d.

Results of SEM observations and EDS graphs of hardened pastes of CEMI42.5MP6P and CEMI42.5NBP20P at 7th-d and 28th-d and 90th-d are presented in Figures 7 and 8. It was observed that in hydration of CEMI42.5MP6P at 7 d, clinker grains were surrounded by radiating fibers of calcium silicate hydrate (C-S-H) resembling pattern of C-S-H of CEMI42.5 cement. Randomly oriented portlandite (CH) crystals and prismatic ettringite crystals were widely dispersed through paste (Figure 7, upper left). However, in CEMI42.5MP6P at the ages of 7 days it was found that marble grains were covered with an amorphous (with respect to C-S-H) layered CH hydration products. Matrix phase is mainly composed of short radicular outgrowths of C-S-Hs around clinker grains and needle-shaped ettringite crystals (Figure 7, upper left). Microstructure of hydrated paste of CEMI42.5MP6P at the ages of 28 days was presented by amorphous gel filling spaces between hydrated particles. In pastes of CEMI42.5MP6P, layered accumulations of CH crystals of about 10 *μ*m in width are intermingled through paste (Figure 7, middle left). There is a visible densification around marble grains due to partial hydration of marble grains, leading to formation of additional CH. At the ages of 90 days marble grains were well located in matrix and were sunk in a layered CH. Observation of paste based on CEMI42.5MP6P demonstrated that marble grains were turned out amorphous reaction prisms. In CH phase, matrix of CEMI42.5MP6P is found to be richer in calcium than that of reference cement paste. At 90th-day, microstructure of CEMI42.5MP6P was further densified with respect to reference cement paste (Figure 7, lower left).

However, compressive strength of CEMI42.5MP6M samples at whole cure periods is notably lower than that of brick powder-blended cement mortar (BP-BCM) and reference cement mortar (RCM). This can be explained by the difference at degree of hydration that occurs in each of two compounds such as CH and C-S-H. A lower degree of hydration occurs in cement paste with 6% MP, where a number of hydration compounds are spotted during SEM imaging, shown in Figure 7. EDS analysis also supported that they were CH, since they exhibited Ca/Si ratios of between 1.76 and 2.8 at 7 d, 28 d, and 90 d. In comparison, hydrates exhibited none traces of carbon at whole periods. Therefore, this low compressive strength exhibited by CEMI42.5MP6M samples may be attributed to increasing of Ca/Si ratio although MP is aiding in densifying microstructure in cement systems by accelerating hydration (Figure 7).

For CEMI42.5BP20P at the ages of 7 days it was found that brick grains were covered with a numerous hydration products of C-S-H gel, layered CH, prism ettringite, and little hollow. Matrix phase of CEMI42.5BP20P is mainly composed of short fibrous C-S-H forming around clinker grains and needle-shaped ettringite crystals, and randomly oriented portlandite (CH) crystals are widely dispersed through paste (Figure 8, upper left). Microstructure of hydrated paste of CEMI42.5BP20P at the ages of 28 days was presented by amorphous gel filling hollow between hydrated particles; this gave certain stability to paste structure. In CEMI42.5BP20P pastes, layered accumulations of C-S-H crystals of about 3 *μ*m in width are intermingled through paste (Figure 8, middle left). There is a visible densification around brick grains due to partial hydration leading to formation of additional C-S-H. At the ages of 90 days, brick grains were well dispersed through matrix. And they penetrated in a layered CH in order to convert CH to fibrous C-S-H forming gel. Observation of paste based on CEMI42.5BP20P demonstrated that brick grains were turned out fibrous reaction prisms. In C-S-H phase, matrix of CEMI42.5BP20P was found to be richer in silicate with respect to reference cement at 90th-day (Figure 8, lower left).

However, compressive strengths of CEMI42.5BP20M samples at whole cure periods were the greatest in these mortars. This can be explained by difference at degree of hydration that occurs in each of one compound as C-S-H. A high degree of hydration occurs in cement paste with 20% BP, where a number of hydration compounds are spotted during SEM imaging, shown in Figure 8. EDS analysis also supported that they were silicate, since they exhibited Si/Ca ratios of about 0.53, 0.63, and 0.96 at 7 d, 28 d, and 90 d, respectively. In comparison, hydrates exhibited none traces of carbon at whole period. Therefore, the highest compressive strength exhibited by CEMI42.5BP20M samples may be attributed to increase of Si/Ca ratio at 90th-day although BP was aiding in densifying microstructure in cement systems by accelerating strength gain slowly (Figure 8).

### 3.2. Influences of the CCC-BCP to CS and FS of Blended Cement Mortars

7th-day, 28th-day, and 90th-day LOI and chemical properties of MP-blended cement pastes and BP-blended cement pastes and CEMI42.5N cement pastes are presented in Tables 4, 5, and 6, respectively.  Table 7 shows 7th-day, 28th-day, and 90th-day CS and FS of MP-blended mortars and BP-blended mortars and CEMI42.5N cement mortars [1].

According to the literature, results showed that mortars containing cement with high LOI were slower strength gains than that of mortars containing cement with low LOI. An increase in LOI of 1% can be anticipated to reduce mortar strength by nearly 4 MPa [20–22, 24, 29–33]. A parallel result was clearly observed in this research due to MP increased LOI in cement and cement pastes, too. As MP was increased up to 35% in cement paste by mass, averages of CaO, MgO, K_2_O, and LOI of MP-blended cement pastes (MP-BCP) at 7 d were, respectively, over 11%, 1%, 9%, and 15% higher with respect to reference paste (RP); averages of SiO_2_, Al_2_O_3_, Fe_2_O_3_, SO_3_, Na_2_O, and alkali of MP-BCP at 7 d were over 20%, 26%, 34%, 29%, 61%, and 46% less than that of RP. Changes of chemical compositions of MP-blended cement pastes (MP-BCPCC) at 7 d caused the decrease of both CS and FS of MP-blended mortars (MP-BM). CS and FS of MP-BM were, respectively, over 23%, 7.5 MPa and 12%, 0.6 MPa less than that of reference mortars (RM) at 7 d, except FS of CEMI42.5MP6M. It was surprisingly detected 0.03 MPa greater FS of CEMI42.5MP6M in comparison with RM although CEMI42.5MP6P oxides changed such as other MP-blended cement pastes at 7 d (Tables 3 and 6). Since MP was increased from 6% to 35% (over 5.5 times) in cement pastes, increasing of MP created 30% CS and 24% FS reduction associated with increasing of 9% CaO, 46% SO_3_, 37% K_2_O, and 33% LOI at MP-BM at 7 d. In view of these changes of MP-BCPCC, CS and FS of MP-BM at 7 d were located in relatively wide ranges of 21.8–31.7 MPa and 4.1–5.4 MPa. These values are the lowest at MP-BMs. Increasing of MP in cement pastes caused a delay at strength of MP-BM at later ages, nevertheless, MP showed the most pronounced enhancing effect on strength gain at early ages: for 20% MP mix at 28 d, CS and FS could be improved by as much as 40% and 12% with respect to MP-BM at 7 d, respectively, (Tables 4 and 7).

Similar strength reduction happened in MP-BM at 7 d was monitored at BP-BM. Since BP was augmented up to 35% in cement paste by mass, BP decreased CaO, MgO, Na_2_O, LOI, and alkali over 3%, 37%, 56%, 8%, and 26% lesser and also increased SiO_2_, Al_2_O_3_, Fe_2_O_3_, SO_3_, and K_2_O over 11%, 30%, 21%, 14%, and 88% higher in comparison with RP at 7 d. Changes of chemical compositions of BP-blended cement paste (BP-BCPCC) resulted reducing both CS and FS of BP-blended mortars (BP-BM). CS and FS of BP-BM were, respectively, over 2%, 0.8 MPa and 4%, 0.2 MPa lower than that of RM at 7 d, except CS of CEMI42.5BP6M and both CS and FS of CEMI42.5BP20M (Tables 3 and 6) [20–22, 24, 32, 33]. It was monitored that CEMI42.5BP20M had the greatest CS and FS in these mortars at 7 d. Result infers that BP has got over 9% greater acceleration effect on strength gain with respect to RM at 7 d. Nevertheless, this effect is not as high as blended mortars containing 20% MP at 28 d. As BP was increased from 6% to 35% (over 5.5 times) in cement pastes, the increasing of BP originated 25% CS and 10% FS reduction accompanying with increasing of 69% Al_2_O_3_, 52% Fe_2_O_3_, 94% MgO, 75% SO_3_, 220% K_2_O, and 25% Na_2_O at BP-BM at 7 d. In view of these changes of BP-BCPCC, CS and FS of BP-BM at 7 d were located in relatively wide ranges of 27.7–37.5 MPa and 4.6–6 MPa. These values are the lowest in BP-BM. However, BP showed the least pronounced enhancing effect on strength gain at early ages: for 20% BP mix at 28 d, CS and FS were, respectively, improved by as much as 23% and 15% in comparison with 7 d (Tables 4 and 7).

It was shown in the literature that an increase in alkali of pastes reduced strength gain by nearly 1.5 MPa at 28 d [20–22, 24, 29–33]. Strength reduction was not monitored in these research results as alkali did not escalate in MP-BCP at 28 d. As MP was escalated up to 35% in cement paste by mass, averages of MgO, SO_3_, and LOI of MP-BCP at 28 d were, respectively, over 60%, 4%, and 11% greater than that of RP; averages of CaO, SiO_2_, Al_2_O_3_, Fe_2_O_3_, K_2_O, and Na_2_O and alkali of MP-BCP at 28 d were, respectively, over 0.5%, 12%, 14%, 13%, 30%, 2%, and 10% less than that of RP. Changes of MP-BCPCC at 28 d reasoned the reduction of both CS and FS of MP-BM. CS and FS of MP-BM were approximately over 24%, 9.5 MPa and 17%, 1.05 MPa less than that of RM at 28 d, except CEMI42.5MP6M (Tables 5 and 7). It was detected that MP-BCPCCC caused over 26% and 9% greater CS and FS at 28 d than that of MP-BMs at 7 d. These results infer that strength gain of MP-BM deals with alkali reduction of MP-BCPCC at 28 d. Since MP was increased from 6% to 35% (over 5.5 times) in cement, this increasing brought about decreasing of 44% CS and 34% FS associated with increasing of 200% MgO and 32% LOI at MP-BMs at 28 d. In view of these changes of MP-BCPCC, CS and FS of MP-BM at 28 d were located in relatively wide ranges of 22.5–40.7 MPa and 4.1–6.2 MPa. These values are higher than those of CS and FS at 7 d (Tables 5 and 7).

Strength reduction accompanying with increasing of alkali for BP-BCP was not observed at BP-BMs at 28 d [20–22, 24, 32]. Strengths augmented nearly 0.5 MPa at BP-BMs although alkali increased over 37% greater with respect to RP. Similar increase at SiO_2_, Al_2_O_3_, Fe_2_O_3_, SO_3_, K_2_O, and Na_2_O associated with increasing of BP up to 35% was, respectively, over 19%, 43%, 49%, 95%, 11%, and 53% greater than that of RP. Decreasing at CaO, MgO, and LOI in BP-BCP was, respectively, over 13%, 1%, and 14% less with respect to RP at 28 d. Changes of BP-BCPCC caused growth of both CS and FS of BP-BM. CS and FS of BP-BM were over 1%, 0.4 MPa and 8%, 0.5 MPa greater in comparison with RM at 28 d (Tables 5 and 7). It was monitored that BP-BCPCCC caused over 30% greater CS and FS at BP-BMs at 28 d than that of BP-BMs at 7 d. It is clear that strength gain of BP-BM deals with reduction of CaO, MgO, and LOI of BP-BCPCC. Since BP was increased from 6% to 35% (over 5.5 times) in cement pastes; this increasing created 3% CS reduction and 13% FS gain associated with increasing of 27% SiO_2_, 36% Al_2_O_3_, 80% Fe_2_O_3_, 67% SO_3_, and 30% Na_2_O at BP-BMs at 28 d. In view of these changes of BP-BCPCC, CS and FS of BP-BM at 28 d were located in relatively wide ranges of 41–46.4 MPa and 6.3–7.5 MPa. These values are lower in comparison with CS and FS of BP-BMs at 90 d (Tables 5 and 7).

Since MP was escalated up to 35% in cement paste by mass, averages of CaO, Na_2_O, LOI, and alkali of MP-BCP were, respectively, over 2%, 262%, 15%, and 89% greater than that of RP at 90 d; averages of SiO_2_, Al_2_O_3_, Fe_2_O_3_, MgO, SO_3_, and K_2_O of MP-BCP were, respectively, over 15%, 13%, 17%, 15%, 26%, and 10% less than that of RP at 90 d [29–31, 33]. Changes of MP-BCPCC at 90 d originated reducing both CS and FS of MP-BM. CS and FS of MP-BM were over 27%, 12 MPa and 4%, 0.2 MPa less than that of RM at 90 d (Tables 6 and 7). Since MP was increased from 6% to 35% in cement pastes, this increasing reasoned reduction of 48% CS and 39% FS associated with increasing of 1% CaO, 44% SO_3_, 7% alkali, and 22% LOI at MP-BM at 90 d. In view of these changes of MP-BCPCC, CS and FS of MP-BM at 90 d were located in relatively wide ranges of 22–42.6 MPa and 3.8–6.5 MPa. These values are the highest in MP-BMs. However, enhancing effect of MP on strength gain gradually decreased over time and after 3 months CS of CEMI42.5MP35 mortar was lesser than that of both CEMI42.5MP6 and RM at 7 d. This implies that MP blending more than 6% has not got positive effect on strength gain of cement mortars at later ages according to these findings of MP-BCPCCC and of MP-BM strength gain in this research (Tables 6 and 7).

On the contrary to last results regarding MP-BCPCCC, as BP was increased up to 35% in cement paste by mass, averages of SiO_2_, Al_2_O_3_, Fe_2_O_3_, K_2_O, Na_2_O, and alkali of BP-BCP were, respectively, over 40%, 53%, 42%, 470%, 313%, and 400% greater in comparison with RP at 90 d; averages of CaO, MgO, SO_3_, and LOI of BP-BCP were, respectively, over 11%, 9%, 19%, and 18% less than that of RP at 90 d. Changes of BP-BCPCCC at 90 d caused ascent of both CS and FS of BP-BM. CS and FS of BP-BM were over 4%, 1.5 MPa and 44%, 2.33 MPa greater than that of RM at 90 d. As BP was increased from 6% to 35% in cement, this increasing caused increasing of 20% CS and 26% FS associated with increasing of 53% SiO_2_, 37% Al_2_O_3_, 60% Fe_2_O_3_, and 6% SO_3_ at BP-BM at 90 d. In view of these changes of BP-BCPCC, CS and FS of BP-BM at 90 d were located in a relatively broad ranges of 42.7–51.7 MPa and 6.5–8.3 MPa. These values are the greatest in CS and FS values in BP-BMs. Moreover, enhancing effect of BP on strength gain gradually increased over time and after 3 months; CS and FS of CEMI42.5BP35 mortars were greater than that of both CEMI42.5BP6 and RM at 7 d. This infers that BP has positive effect on strength gain of cement mortars at later ages (Tables 6 and 7).

If changes of 90th-day MP-BCPCC are considered as final results, it could be generalized that effects of MP-BCPCCC on strength gain deal with fluctuation of SiO_2_, Na_2_O and alkali content. Similar ups and downs of SiO_2_, Na_2_O and alkali at MP-BCP are observed CS and FS gain at MP-BM as a bumpy characteristic. Both averages of 7th-day compositions and averages of 7th-day strengths were lower in comparison with 28th-day and were higher with respect to 90th-day as shown in the following parentheses (7 d: 14.11% SiO_2_, 0.24% Na_2_O, 0.422% alkali, 26.31 MPa CS and 4.75 MPa FS; 28 d: 14.76% SiO_2_, 0.29% Na_2_O, 0.425% alkali, 33.4 MPa CS and 5.2 MPa FS; 90 d: 12.84% SiO_2_, 0.10% Na_2_O, 0.13% alkali, 31.8 MPa CS, and 5.17 MPa FS). And effect of BP-BCPCCC on CS and FS gaining deals with continuously increasing SiO_2_. Similar upward movement of SiO_2_ at BP-BCP is monitored CS and FS gain at BP-BM. Both compositions and strengths develop progressively as seen in the following parentheses (7 d: 19.59% SiO_2_, 33.45 MPa CS and 5.16 MPa FS; 28 d: 20.03% SiO_2_, 43.53 MPa CS, and 6.76 MPa FS; 90 d: 21.12% SiO_2_, 45.73 MPa CS and 7.77 MPa FS) (Tables 4, 5, 6, and 7).

## 4. Conclusions

This research was carried out to more effectively explain CS and FS gains associated with BCP-CCC. Chemical compositions of MP-BCP and BP-BCP and CS and FS of MP-BM and BP-BM were determined by international standards at 7th-day, 28th-day, and 90th-day. The results of these experiments support the following conclusions.

1. At early ages of hardening, CEMI42.5MP6P has a microstructure composed of the gelatinous membrane around the marble powders, layered CH formations, amorphous C-S-H gel, and ettringite prisms. At the age of 28 days and 90 days, further encapsulation of marble powders into hydration products is observed. However, at the ages of 7 days, CEMI42.5BP20P has a microstructure composed of fibrous C-S-H gel, layered CH formations, and ettringite prisms. At the age of 28 days and 90 days, further encapsulation of brick powders into hydration products is observed. Compressive strength of the CEMI42.5MP6M samples at whole cure periods is notably lower than that of BP-BCM and RCM samples. This can be explained by the increase of Ca/Si ratio from 1.76 up to 2.8. On the contrary to last conclusion, compressive strength of the CEMI42.5BP20M is the greatest in the mortars. This can be explained by the increase of Si/Ca ratios from 0.53 up to 0.96.
2. MP-blended cement pastes displayed increasing at CaO, MgO, K_2_O and LOI and reduction at SiO_2_, Al_2_O_3_, Fe_2_O_3_, MgO, SO_3_, K_2_O and Na_2_O at 7 d; ascent at MgO, SO_3_ and LOI and reduction at CaO, SiO_2_, Al_2_O_3_, Fe_2_O_3_, K_2_O and Na_2_O at 28 d; and increasing at CaO, Na_2_O, LOI and alkali and reduction at SiO_2_, Al_2_O_3_, Fe_2_O_3_, MgO, SO_3_, K_2_O at 90 d. Regardless of the increasing presence of CS and FS at CEMI42.5BP20M, MP blending up to 6% showed the highest mortar CS and FS in the presence of MP-BCPCCC at 7 d. This result infers that small blending of MP for cement had positive effect on strength gains of mortars at early ages. At high blending levels up to 35% with this method being applied in this research, none of MP percent had a greatly positive effect on strength gain at early and later ages. Increasing of LOI and alkali at MP-BCP caused reduction of strength gain at MP-BM according to the literature. However, effect of MP-BCPCCC on strength gain of MP-BM deals with fluctuation of SiO_2_, Na_2_O, and alkali as well as increasing of LOI. Strength gain of MP-BM fluctuates because of *vicissitudes* of SiO_2_, Na_2_O, and alkali content at MP-BCP. This result showed that MP blending more than 6% had no positive effect on strength gains of mortars at early ages.
3. On the contrary to effects of MP-BCPCCC on strength gain, BP-blended cement pastes displayed reduction at CaO, MgO, Na_2_O, LOI and alkali, and ascent at SiO_2_, Al_2_O_3_, Fe_2_O_3_, SO_3_, and K_2_O at 7 d; decreasing at CaO, MgO and LOI and ascent at SiO_2_, Al_2_O_3_, Fe_2_O_3_, SO_3_, and K_2_O, Na_2_O and alkali at 28 d; and reducing at CaO, MgO, SO_3_ and LOI of and increasing at SiO_2_, Al_2_O_3_, Fe_2_O_3_, K_2_O, Na_2_O and alkali at 90 d. Blending of BP accelerated tendency of strength gain at later ages because BP reduced LOI in the BP-BCPCC. CEMI42.5BP20M displayed the greatest CS and FS in the mortars at whole ages. Effects of BP-BCPCCCs on CS and FS gains deals with increasing of SiO_2_ in BP-BCP. Average of SiO_2_ tended to increase in BP-BCP continuously. Strength gain at BP-BM acted similar to SiO_2_ in BP-BCP. Since CEMI42.5BP20M achieved nearly 52.5 MPa compressive strength at 90 d, BP blending up to 35% had a positive effect on strength gains of cement mortars at later ages.

## Figures and Tables

**Table 1:** Physical and pozzolanic properties of MP and BP and physical properties of CEMI42.5N cement [1].

Physical Properties	MP	BP	CEMI42.5N
Fineness, (per cent)			
40 *µ*m	9.05	28.27	9.5
90 *µ*m	3.58	5.85	0.3
200 *µ*m	1.34	0.23	0.1
Density (gr/cm^3^)	2.53	2.12	3.11
Specific surface (Blaine method) (m^2^/kg)	620.8	597.8	325
Water permeability (%)	16.35	40.10	—
Pozzolanic properties	MP	BP	CEMI42.5N
Compressive strength (CS) (MPa)	1.15	17.06	—
Flexural strength (FS) (MPa)	1.03	3.7	—

**Table 2:** Codes of cement samples and mixture proportions and grinding time [1].

Codes of cements	Mixture proportions (%)				Grinding time (min)
	MW	BW	Clinker CEMI42.5N	Gypsum	
CLINKER CEMI42.5MP6	6	0	89	5	30
CLINKER CEMI42.5MP20	20	0	75	5	30
CLINKER CEMI42.5MP21	21	0	74	5	30
CLINKER CEMI42.5MP35	35	0	60	5	30
CLINKER CEMI42.5BP6	0	6	89	5	30
CLINKER CEMI42.5BP20	0	20	75	5	30
CLINKER CEMI42.5BP21	0	21	74	5	30
CLINKER CEMI42.5BP35	0	35	60	5	30
CEMI42.5N (Reference)	0	0	95	5	30

**Table 3:** Codes of blended cement mortars, reference cement mortars, and mixture proportions for standard 3-gang mould Kırgız, 2007 [1].

Codes of cements	Mixture proportions for standard 3-gang mould					Water-binder ratio
	CEMI42.5N (g)	MP (g)	BP (g)	Sand (g)	Water (mL)	
CEMI42.5MP6M	423	27	0	1350	225	0.5
CEMI42.5MP20M	360	90	0	1350	225	0.5
CEMI42.5MP21M	355.5	94.5	0	1350	225	0.5
CEMI42.5MP35M	292.5	157.5	0	1350	225	0.5
CEMI42.5BP6M	423	0	27	1350	225	0.5
CEMI42.5BP20M	360	0	90	1350	225	0.5
CEMI42.5BP21M	355.5	0	94.5	1350	225	0.5
CEMI42.5BP35M	292.5	0	157.5	1350	225	0.5
CEMI42.5NM (Reference)	450	0	0	1350	225	0.5

**Table 4:** 7th-day chemical compositions of marble and brick powder-blended and CEMI42.5N cement paste samples.

Codes of pastes	7th-day chemical compositions (%)									
	CaO	SiO_2_	Al_2_O_3_	Fe_2_O_3_	MgO	SO_3_	K_2_O	Na_2_O	LOI	Alkali
CEMI42.5MP6P	49.35	16.22	4.34	3.35	6.38	2.40	0.24	0.33	17.16	0.49
CEMI42.5MP20P	54.65	14.34	3.26	3.36	1.84	2.35	0.22	0.20	19.03	0.35
CEMI42.5MP21P	55.59	14.30	3.06	2.34	2.08	2.65	0.30	0.18	19.10	0.38
CEMI42.5MP35P	54.24	11.60	2.98	2.34	1.65	3.52	0.33	0.25	22.95	0.47
CEMI42.5BP6P	52.36	17.26	4.10	4.13	1.07	3.24	0.25	0.24	17.23	0.41
CEMI42.5BP20P	46.44	19.98	6.29	5.24	1.45	3.84	0.63	0.29	15.62	0.71
CEMI42.5BP21P	44.36	19.36	6.48	5.17	2.86	4.80	0.45	0.25	15.59	0.55
CEMI42.5BP35P	42.67	21.78	6.94	6.29	2.08	5.70	0.55	0.30	13.67	0.66
CEMI42.5NP (Reference)	48.12	17.68	4.59	4.29	2.95	3.87	0.25	0.61	16.95	0.78

**Table 5:** 28th-day chemical compositions of marble and brick powder-blended and CEMI42.5N cement paste samples.

Codes of pastes	28th-day chemical compositions (%)									
	CaO	SiO_2_	Al_2_O_3_	Fe_2_O_3_	MgO	SO_3_	K_2_O	Na_2_O	LOI	Alkali
CEMI42.5MP6P	53.37	16.56	3.61	3.47	1.31	2.73	0.22	0.33	18.03	0.48
CEMI42.5MP20P	52.50	14.25	3.44	2.57	2.85	2.22	0.15	0.20	21.01	0.30
CEMI42.5MP21P	52.62	15.21	3.49	2.78	1.50	3.01	0.25	0.43	20.25	0.60
CEMI42.5MP35P	51.69	13.02	3.07	2.40	2.66	1.85	0.15	0.22	23.95	0.32
CEMI42.5BP6P	48.59	17.96	4.64	3.45	2.80	3.50	0.30	0.42	17.45	0.62
CEMI42.5BP20P	46.85	19.86	5.97	4.55	0.73	4.35	0.33	0.45	16.32	0.67
CEMI42.5BP21P	47.27	19.46	5.89	5.04	0.41	4.69	0.25	0.41	15.83	0.58
CEMI42.5BP35P	41.11	22.85	6.33	6.22	1.21	5.85	0.27	0.55	14.72	0.73
CEMI42.5NP (Reference)	52.84	16.72	3.97	3.21	1.30	2.35	0.26	0.30	18.67	0.47

**Table 6:** 90th-day chemical compositions of marble and brick powder-blended and CEMI42.5N cement paste samples.

Codes of pastes	90th-day chemical compositions (%)									
	CaO	SiO_2_	Al_2_O_3_	Fe_2_O_3_	MgO	SO_3_	K_2_O	Na_2_O	LOI	Alkali
CEMI42.5MP6P	51.69	14.92	4.05	3.07	2.23	2.26	0.03	0.11	21.34	0.13
CEMI42.5MP20P	52.56	12.84	3.67	2.68	1.98	2.37	0.06	0.11	23.18	0.15
CEMI42.5MP21P	51.69	12.76	3.55	2.63	1.45	3.29	0.03	0.09	24.31	0.11
CEMI42.5MP35P	52.29	10.84	2.69	2.87	1.75	3.26	0.05	0.11	26.01	0.14
CEMI42.5BP6P	49.87	16.78	5.14	3.64	1.98	3.50	0.08	0.09	18.49	0.14
CEMI42.5BP20P	45.63	19.36	6.24	4.98	2.47	1.99	0.24	0.14	18.36	0.30
CEMI42.5BP21P	44.96	22.52	6.13	4.93	1.74	2.98	0.22	0.09	16.23	0.24
CEMI42.5BP35P	40.99	25.82	7.08	5.80	1.79	3.74	0.40	0.18	13.98	0.44
CEMI42.5NP (Reference)	50.88	15.06	3.99	3.38	2.17	3.75	0.05	0.04	20.57	0.07

**Table 7:** 7th-day and 28th-day and 90th-day CS and FS of marble and brick powder-blended and CEMI42.5N cement mortar samples [1].

Codes of mortars	Compressive strength (MPa)			Flexure strength (MPa)		
	7th-day	28th-day	90th-day	7th-day	28th-day	90th-day
CEMI42.5MP6M	31.76	40.72	42.61	5.40	6.26	6.37
CEMI42.5MP20M	26.99	37.82	32.31	4.84	5.43	5.30
CEMI42.5MP21M	24.61	32.52	30.26	4.66	5.00	5.13
CEMI42.5MP35M	21.89	22.55	22.04	4.10	4.12	3.88
CEMI42.5BP6M	37.35	42.74	42.76	5.26	6.37	6.59
CEMI42.5BP20M	37.53	46.41	51.73	6.09	7.04	8.60
CEMI42.5BP21M	31.18	43.92	44.08	4.62	6.43	7.59
CEMI42.5BP35M	27.76	41.05	44.36	4.70	7.21	8.32
CEMI42.5NM (Reference)	31.76	40.72	42.61	5.40	6.26	6.37
